# Chlamydia trachomatis enhances HPV persistence through immune modulation

**DOI:** 10.1186/s12879-024-09094-6

**Published:** 2024-02-20

**Authors:** Yingying Lu, Qi Wu, Li Wang, Lingting Ji

**Affiliations:** 1 Department of Clinical Laboratory, Shanghai Seventh People’s Hospital , Shanghai University of Traditional Chinese Medicine, Shanghai, 200199 China; 2 Department of Clinical Laboratory, Shanghai Zhabei Central Hospital, Shanghai, 200070 China

**Keywords:** Chlamydia trachomatis, HPV, Langerhans cell, Pathway, Immune suppression

## Abstract

*Chlamydia trachomatis* (CT) is the most common sexually transmitted infections globally, and CT infection can enhance HPV persistence. Epidemiological analysis has shown that patients with CT/HPV coinfection have a higher risk of developing cervical cancer and exhibit more rapid progression to cervical cancer than patients with HPV infection alone. However, the mechanism has not been fully elucidated. Here, we report that CT infection supports HPV persistence by further suppressing the functions of Langerhans cells (LCs); in particular, CT further activates the PI3K pathway and inhibits the MAPK pathways in LCs, and these pathways are frequently involved in the regulation of immune responses. CT/HPV coinfection also impairs LC functions by reducing the antigen-presenting ability and density of LCs. Moreover, CT/HPV coinfection can alter T-cell subsets, resulting in fewer CD4 + and CD8 + T cells and more infiltrating Tregs. Moreover, CT/HPV coinfection decreases the CD4 + /CD8 + T cell ratio to below 1, coinfection also induces greater T lymphocytes’ apoptosis than HPV infection, thus impairing cell-mediated immunity and accelerating the progress to cervical cancer.

**Supplementary Information:**

The online version contains supplementary material available at 10.1186/s12879-024-09094-6.

## Introductions

Cervical cancer is the fourth most common malignant tumour in women worldwide [1]. There were approximately 106,200 new cases in China in 2018, ranking first in the world [2], and the disease continues to be a major public health concern.

Persistent infection with high-risk HPV genotypes (hr-HPVs) is recognized as the most important risk factor for the development of cervical cancer [3]. The evidence that CT may decrease the efficient clearance of HPV infection, favouring HPV persistence, is particularly interesting [4, 5].

HPV is the most common sexually transmitted infections globally [6]. It’s usually coinfected with *Chlamydia trachomatis.*The partnership between HPV and *C. trachomatis* is basically due to their similarities: common transmission routes, reciprocal advantages, and the same risk factors.

According to World Health Organization (WHO) estimates, approximately 290 and 131 million new cases of HPV and CT respectively, occur each year [7, 8]. The coinfection rates of HPV and CT differ in different regions. In southern Hunan Province in China, the incidence is 1.2% [9],while the CT/HPV coinfection rate is 2.7% in Italian females with normal cytology [10]. The natural history of this coinfection is strongly conditioned by the balance between the host microbiome and immune condition and the infecting agent.

CT has been reported to interfere with viral clearance, supporting the persistence of HPV infection. Two potential mechanisms have been proposed: CT infection may damage the mucosal barrier and promote the entry of HPV**,** or it may impair the immune response, favouring the persistence of HPV [5, 11]. A meta-analysis confirmed that patients with CT/HPV coinfection have a higher risk of developing cervical cancer and exhibit more rapid progression to cervical cancer [12]. Recently, Abdul Khan reported that CT coinfection with HPV may modulate cervical cancer development [13].

HPV can achieve persistent infection mainly because it has evolved immune evasion capacity by suppressing the function of Langerhans cells (LCs) [14, 15] and subsequently affects the immune response. LCs are local antigen-presenting cells (APCs) in the epithelium that specialize in the uptake, processing, and presentation of antigens to T cells and induce both CD8 + and CD4 + T cell responses [16]. LCs are responsible for initiating adaptive immune responses against pathogens that enter the epithelial layer.

When encountering pathogenic antigens, LCs become activated by phenotypic and functional changes, including the activation of signalling cascades, the increased expression of costimulatory molecules and the secretion of inflammatory cytokines. Activated LCs then travel to lymph nodes to cross-present HPV peptides to T cells and initiate adaptive T-cell responses against the pathogen [17].

However, in HPV infection, LC is less likely to become mature APC but exhibits dysregulation of the PI3K/AKT and MAPK pathways [17]. These two signalling pathways are frequently involved in the regulation of immune responses. After HPV infection, the PI3K signalling cascade is activated, whereas the MAPK pathways are inhibited in LCs. PI3K signalling cascade activation leads to the upregulation of phosphorylated Akt, and Akt plays diverse roles in the regulation of multiple signalling cascades. Akt can activate NF-ĸB, inhibit MAPK pathways and inhibit Iĸ-B kinase [18], thereby regulating multiple signalling pathways that are involved in the control of immune responses.

Previous investigations have shown that inhibition of the PI3K cascade can upregulate surface activation marker expression of LC and induce a potent immune response in HPV infection [19].HPV-induced immunosuppressive LCs are therefore unable to initiate effective cytotoxic T-cell responses against HPV. These data indicate that the activation of the PI3K and MAPK pathways defines a novel immune evasion mechanism of HPV.

Invaded *C.trachomatis* also induces long-lasting activation of the PI3/Akt signaling pathway, which that is required for replication of *C.trachomatis*; however, it is unclear whether MAPK/ERK signalling is regulated by *C. trachomatis* infection [20, 21].

CT interferes with immune responses and allows the persistence of HPV [4, 5]. It has been reported that CT infection can decrease the number of antigen-presenting cells and reduce the antigen-presenting abilities of LCs [22]. CT can also affect CD4 + and CD8 + T cells [23]. However, the role of CT in the process of HPV infection has not been fully elucidated.

The primary objective of this study was to investigate whether CT exacerbates HPV-induced immune suppression by dysregulating the PI3K/AKT and MAPK pathways and inhibiting the antigen-presenting abilities of LCs and to determine whether CT infection can change T-cell subsets and induce apoptosis, thus to define the immune mechanism of CT in enhancing persistent HPV infection and accelerating the progression of cervical cancer.

## Material and methods

### Patient material

In this study, 90 patients with HPV 16 infection (the most common hr-HPV in China) and 30 patients with chronic CT infection alone in Shanghai Zhabei Central Hospital from July 2017 to July 2018 were enrolled. Written informed consent for blood and tissue sampling was obtained from all individuals under a protocol approved by the Institutional Review Board of Shanghai Zhabei Central Hospital.

Among the 90 patients with HPV 16 infection, 37 patients were in the CT/HPV coinfection group, and 53 patients were in the HPV infection group. Thirty patients with CT infection and thirty healthy donors (control group) were also enrolled in the study. The patient characteristics are summarized in Table 1. No statistical significance (*P* > 0.05) was observed in the general information of each group. Blood samples, exfoliated cervical cells and cervical epithelial biopsy samples (2–5 mm^2^) were collected. Exfoliated cells were collected via cervical swabs. HPV genotyping was performed using the INNO-LiPA HPV Genotyping Extra kit (Innogenetics, Seguin, TX). CT was screened using the ABON *Chlamydia trachomatis* antigen detection kit (Fujian, China), and the results were confirmed by the Chlamydia TaqMan PCR Kit (Norgen Biotek, Canada).The cytological results were categorized following the 2014 Bethesda System as (a) negative for an intraepithelial lesion or malignant (NILM), (b) atypical squamous cells of undetermined significance (ASCUS), (c) low-grade squamous intraepithelial lesion (LSIL), (d) atypical squamous cells and high-grade squamous intraepithelial lesion cannot be excluded (ASC-H), (e) high-grade squamous intraepithelial lesion (HSIL), or (f) squamous cell carcinoma (SCC) [23].

**Table 1:** Patient demographics and disease characteristics

Clinical parameter	HPV	CT/HPV	C. trachomatis	Control
No. of patients	53	37	30	30
Median age (range)	36.22 ± 6.17(20–65)	35.86 ± 5.42(21–63)	33.43 ± 4.12(18–60)	34.57 ± 7.29(22–60)
Pathological diagnosis				
Negative	39(73.5%)	22(59.4%)	24(80.0%)	
ASC-US	6(11.3%)	3(8.1%)	3(10.0%)	
LSIL	5(9.4%)*	8(21.6%)	2(6.7%)*	
HSIL	3(5.7%)*	4(10.8%)	1(3.3%)*	
Total	53	37	30	

* *P* < 0.05 compared with CT/HPV coinfection group

### Langerhans cell generation and costimulatory molecule analysis

LCs were separated from the PBMCs of patients in the different groups as previously described [24]. Briefly, PBMCs were incubated in RPMI medium supplemented with 1000 U/ml GM-CSF, 2 mM L-glutamine, 10 mg of streptomycin, 10% foetal bovine serum (FBS), 1000 U/ml IL-4, and 10 μg/ml TGF-β for 7 days. The LC phenotype (CD1a + Langerin + CD14-) was confirmed by flow cytometry.

Surface marker expression was analysed by flow cytometry. Briefly, 10^6^ LCs were harvested, washed, stained with antibodies against MHC I (ab134189, Abcam), MHC II (ab55152, Abcam), CD80 (ab134120, Abcam), CD83 (ab275021, Abcam), CD86 (ab239075, Abcam), and CD40 (ab224639, Abcam Inc., Cambridge, UK) or with isotype controls for 1 h, and washed with FACS buffer. All the flow cytometric analyses were performed using an FC500 flow cytometer. Then, we treated the LCs with 20 μM LY294002 (EMD Biosciences), a potent specific inhibitor of PI3K, and analysed surface marker expression again. The flow cytometric data were quantified as the median fluorescence intensity (MFI) and analysed using FlowJo software (FlowJo, LLC).

### Immunofluorescence staining

The density of Langerhans cells (LCs) and Treg infiltration were measured in cervical biopsy samples. LCs are Langerin (CD207)-positive dendritic cells that constitutively express CD11c [25]. The cervical biopsy samples were cut into frozen sections with a thickness of 5 µm and stained with an anti-CD207Ab(ab283686, Abcam), followed by staining with a TRITC-conjugated secondary antibody. Then, the cells were incubated an anti-CD11c antibody (ab52632, Abcam Inc., Cambridge, UK), followed by a FITC-conjugated secondary antibody. For the detection of Tregs, cervical biopsy samples were stained with an anti-FOXP3 antibody (ab215206, Abcam), followed by a FITC-conjugated secondary antibody.

The samples were imaged by confocal fluorescence microscopy, and cells that were visible as fluorescent objects with a size greater than 4.5 µm were quantified using ImageJ software (National Institute of Mental Health, Bethesda, USA) [26].

### Western blot analysis

LCs from different groups were collected as described above, washed twice with PBS. Cellular extracts were prepared using the Mammalian Protein Extraction Reagent (Pierce). Immunoblotting was performed using Primary antibodies top-ERK1/2(CST,4694S, cell signaling Technology Co.LTD. Massachusett, USA), ERK1/2(CST,4695S), p-MAPK kinase (MKK)4(CST,9151S), MKK4(CST,9152S), PI3K(ab278545), p-PI3K (ab278545,ABCAM Inc.,Cambridge,Uk), AKT(CST,9272S) and p-AKT(CST,13038S) or GAPDH(CST,5174 T, cell signaling Technology Co. LTD. Massachusett,USA).

GAPDH was used as an internal control as previously reported [27]. HRP-conjugated anti-rabbit or anti-mouse secondary antibodies were also used. The proteins were visualized using enhanced chemiluminescence Western blotting detection reagents and detected with an HMIAS-2000 Imaging System. Band densities were determined by Bio-Rad Quantity One software and quantified as the ratio to GAPDH.

### Antigen-presenting ability analysis

Antigen-presenting ability analysis was performed using a mixed lymphocyte response assay (MLR) [28]. Cervical epithelial biopsy samples (2–5 mm^2^) were incubated with Dispase II (2.4 U/ml) for 45 min and then digested with 0.03% trypsin and 0.02% DNase; approximately (0.05–0.2) × 10^6^ epithelial cells were obtained from each sample. Epithelial cells (including 500–1000 LCs) were used as stimulators, and the same numbers of PBMCs from the control group were used as responders. The cells were mixed and incubated in RPMI 1640 for 3 days. [3H]-TdR (0.5 μCi/well) was added for the final 18 h of incubation. The, the cells were harvested, and incorporation of [3H]-TdR into DNA was measured using a Top Count Microplate Scintillation and Luminescence Counter (Packard Instrument, Meriden, CO). The results are expressed as the proliferation index (mean radioactive cpm (mean counts per minute)/mean cpm).

### Flow cytometry

Flow cytometry was used to analyse CD4 + and CD8 + T-cell subsets as well as T lymphocyte apoptosis. PBMCs were separated by using Ficoll density gradient centrifugation. A total of 5 × 10^6^ cells/ml from each patient or healthy donor were separately suspended in PBS and stained for 1 h at 4°C with the following monoclonal antibodies: CD4-PEcy7 (ab233660, Abcam Inc.), CD8-APC-Cy7 (ab233300, Abcam Inc.), and CD3-FITC (ab34275, Abcam Inc., Cambridge, UK). Then, the cells were resuspended in cold PBS. All the samples were analysed using a BD FACS Canto II flow cytometry system (BD Biosciences).

The apoptosis of T lymphocytes was assessed using PE-Annexin V staining (BD Biosciences). The data were analysed using FlowJo software.

### Statistics

SPSS 20.0 statistical software was used to process the statistical data. The statistical significance of the different assays (costimulatory molecule assay, LC density analysis, MLR assay and flow cytometry) was determined by one-way ANOVA for overall significance followed by Tukey's multiple comparisons test. Nonparametric Mann–Whitney U tests were performed to analyse representative experiments of technical replicates from single donors performed in triplicate wells. *P* < 0.05 was considered to indicate significant differences.

## Results

### Expression of antigen presentation and costimulatory molecules on LCs in different groups of patients

**Fig. 1:**
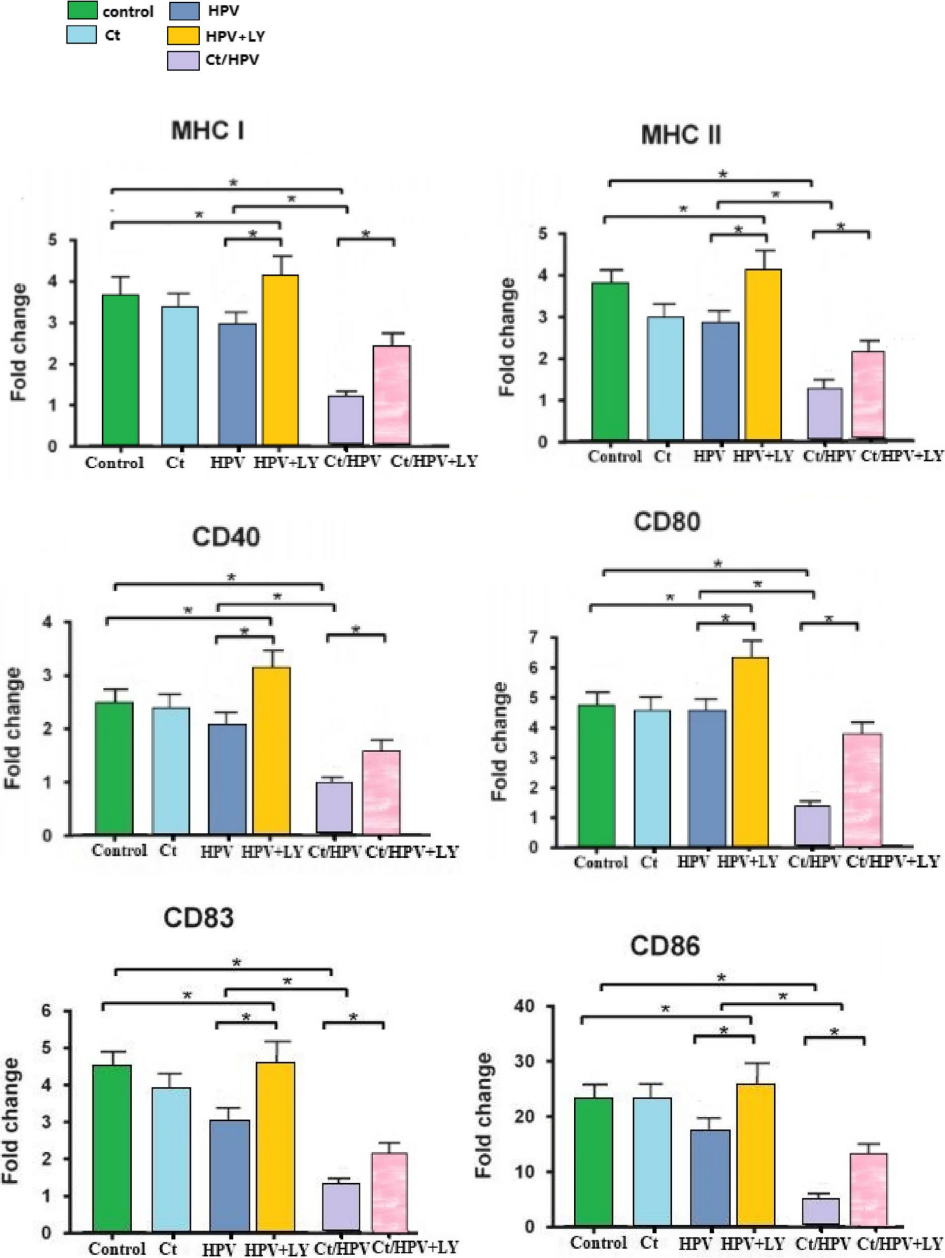
Expression of MHC and costimulatory molecules on LCs from different groups. MHC I, MHC II, CD40, CD80, CD83, and CD86 expression was assessed via flow cytometry. The data are presented as the mean fold change in surface marker expression relative to normal LCs based on mean fluorescence intensity (MFI). (*N* = 30 for each group). *p < 0.05, compared to the control group (one-way ANOVA, Tukey's post test)

LCs expressed low levels of CD40, CD80, CD86 and the maturation marker CD83. The CT infection group had slightly decreased expression of MHC and activation markers compared to the control group. The HPV infection group demonstrated nonsignificant but lower levels of MHC I, MHC II and costimulatory molecules on LCs than the control group (*P* > 0.05), which was consistent with the idea that HPV suppresses LC activation to some extent. In the CT/HPV coinfection group, the expression levels of MHC and costimulatory molecules on LCs were significantly decreased compared to those in the HPV infection group, suggesting that CT potentially suppresses immune responses*.* However, in the HPV infection group, treatment with the PI3K inhibitor LY294002 caused significant upregulation of all the surface markers that were analysed. The results are shown in Fig. 1.

These data show that LCs from the HPV and CT groups did not have notably decreased marker expression, but CT/HPV coinfection significantly suppressed the expression of MHC and costimulatory molecules. However, after treatment with the PI3K inhibitor LY294002 in the HPV and Ct/HPV group, the LCs exhibited significantly increased marker expression (Fig. 1). These data indicate that during CT/HPV coinfection, the suppression of PI3K can upregulate the surface marker expression by LCs.

### HPV infection inhibits MAPK signalling and activates PI3K signalling in LCs

**Fig. 2:**
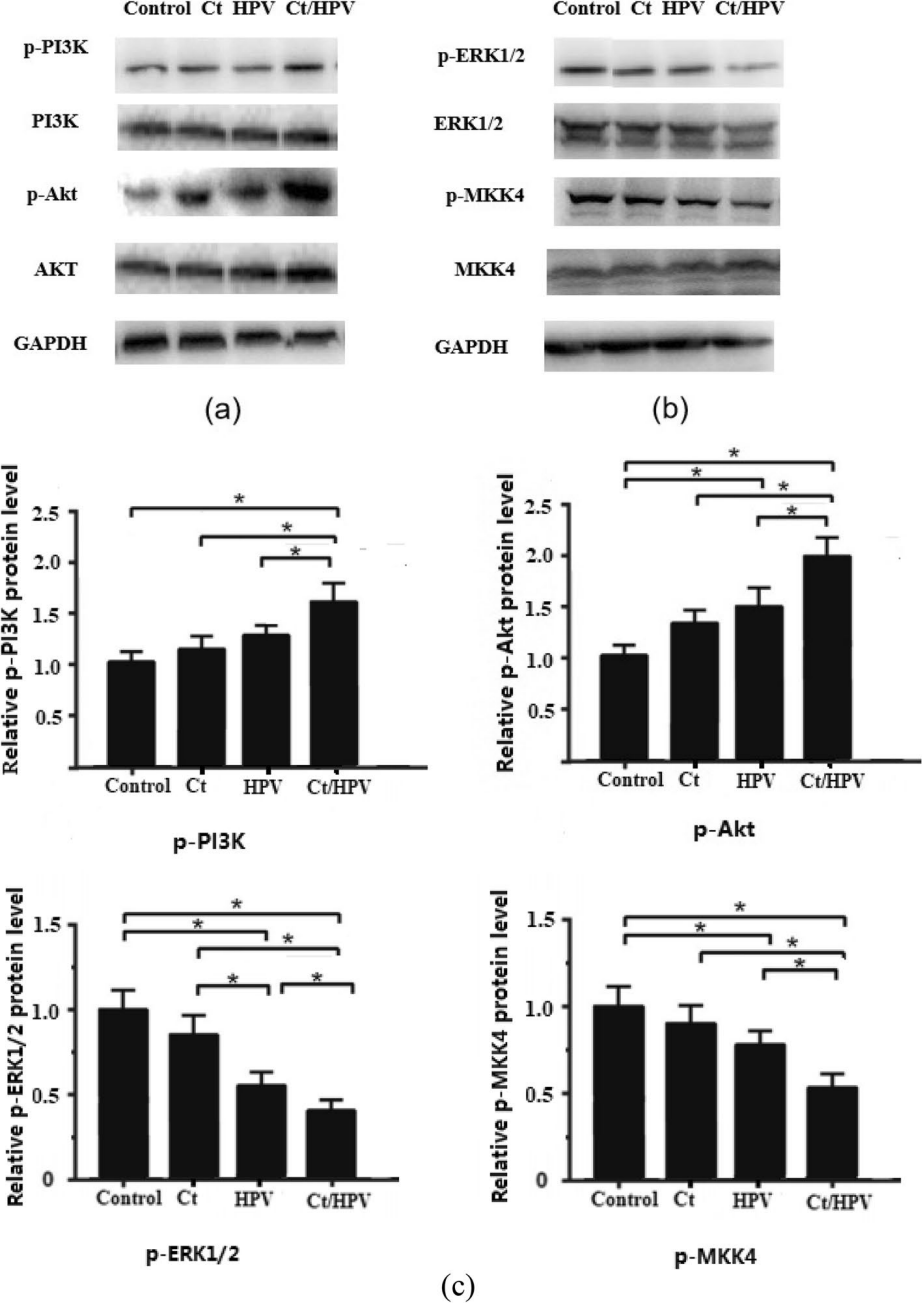
Western blot analysis of the PI3K/Akt and MARK pathways in different groups of patients: (**a**) activation of PI3K by HPV and CT/HPV coinfection (**b**) downregulation of the MAPK pathway in HPV and CT/HPV coinfection (c) The expression of MAPK and PI3K pathway proteins (*N* = 30 for each group). *p < 0.05, compared to another group (one-way ANOVA, Tukey's test). Gels used in figures are compliant with the digital image and integrity policies

Western blot analysis showed that the levels of phosphorylated PI3K and Akt in LCs from the HPV infection and CT infection groups were enhanced compared with those from the control group, and CT/HPV coinfection elevated the phosphorylation of PI3K and Akt compared with HPV infection or CT infection alone (Fig. 2a, c).

MAPK pathways in LCs are suppressed by HPV infection. LCs exhibited decreases in p-ERK1/2 and p-MKK4 in the HPV infection group (Fig. 2b, c) compared with the control group, and in the CT/HPV coinfection group, MAPK pathways were much more suppressed than in the HPV and CT infection groups (*p* < 0.05).

Overall, the data indicate that Ct infection affects the signaling pathways involved in HPV infection, and the further activation of PI3K/Akt and the suppression of MARK pathways by CT/HPV coinfection aggravate the suppression of LCs to induce an immune response.

### Comparison of the antigen-presenting abilities of LCs from patients in different groups

**Fig. 3:**
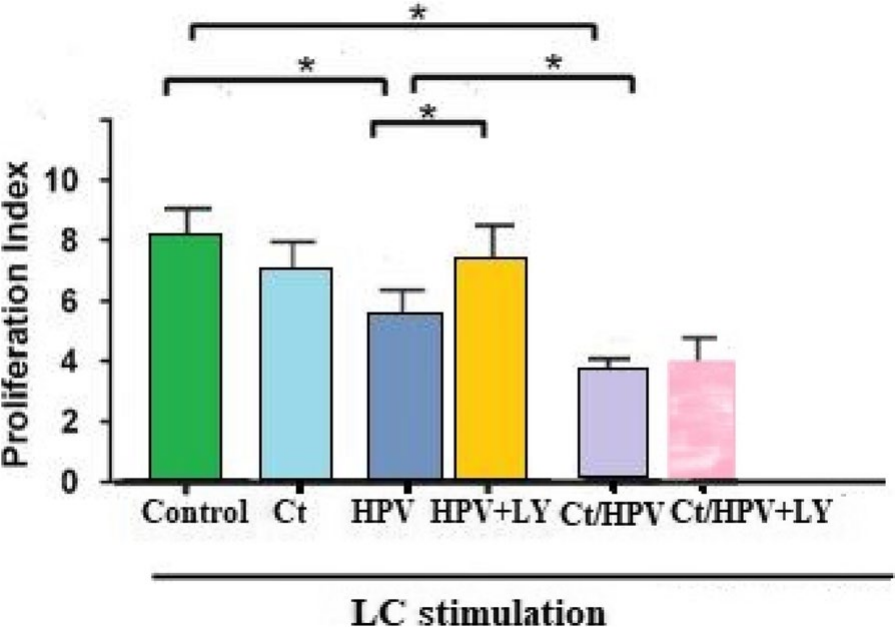
Comparison of the antigen-presenting ability of mixed lymphocytes in different groups (*N* = 30 from each group; differences were determined using one-way ANOVA and Tukey’s test for median comparisons. * represent significant differences (*P* < 0.05)

The antigen-presenting abilities of LCs from patients were analysed by MLR, and the results of different groups were compared; the results are presented as percentage of PBMC proliferation (Fig. 3). It was found that the antigen-presenting abilities of LCs in both the CT/HPV coinfection and HPV infection groups were significantly decreased compared to those in the control group (*P* < 0.05); the CT infection group exhibited decreased antigen-presenting ability compared to the control group, but without significance; and the antigen-presenting ability in the CT/HPV coinfection group was significantly lower than that in the HPV infection group (*P* < 0.05). These results indicate that HPV infection suppressed the function of LCs, and coinfection with CT exacerbated this suppression.When applied with the PI3K inhibitor LY294002 in the HPV infection group, antigen presenting ability incerased significantly;while applied with Ct/HPV group, antigen presenting ability increase without significance.

### Comparison of LC density in different groups

**Fig. 4:**
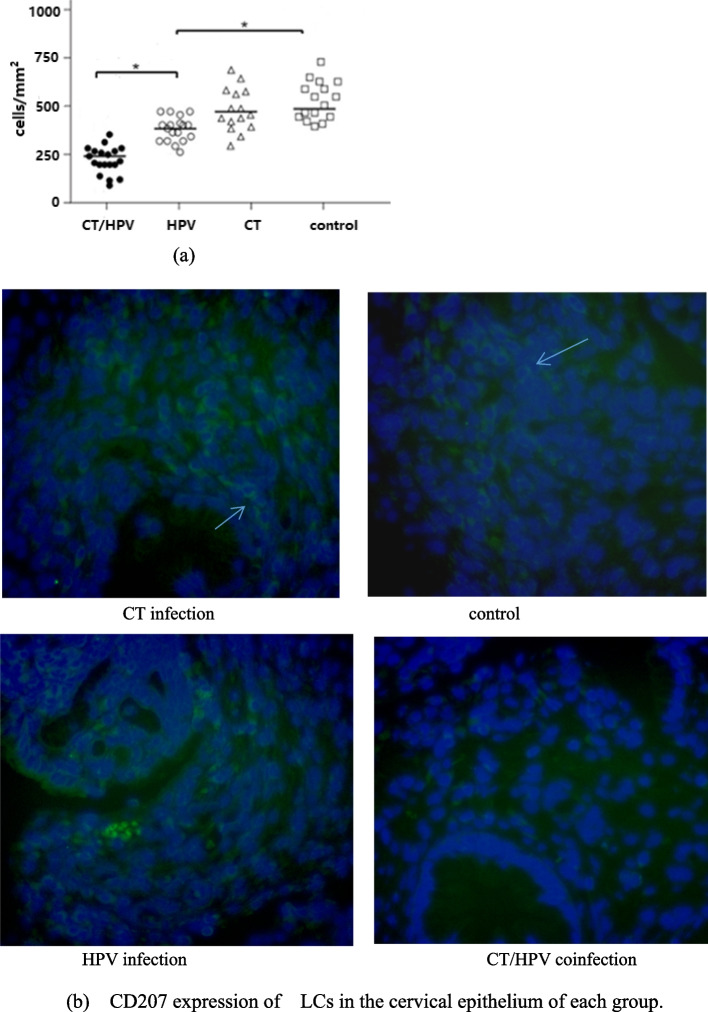
In vivo imaging and quantification of LCs in specimens from each group. **a** Quantification of the cell number in different frames that covered an imaging zone 1 cm in diameter. The results are presented as the mean ± SEM, * represents significant differences (*P* < 0.01). **b** Representative images of Langerhans cells are shown from specimens from each group. The arrow indicates CD207-positive cells

The density of LCs was determined by immunofluorescence staining of CD207. LC density was significantly decreased in the HPV infection group compared with the control group (*P* < 0.01), and LC density was further decreased in the CT/HPV coinfection group, a significant difference exists between the CT/HPV coinfection group the HPV infection group (*P* < 0.01) (Fig. 4).

### Immunofluorescence staining of tregs

**Fig. 5:**
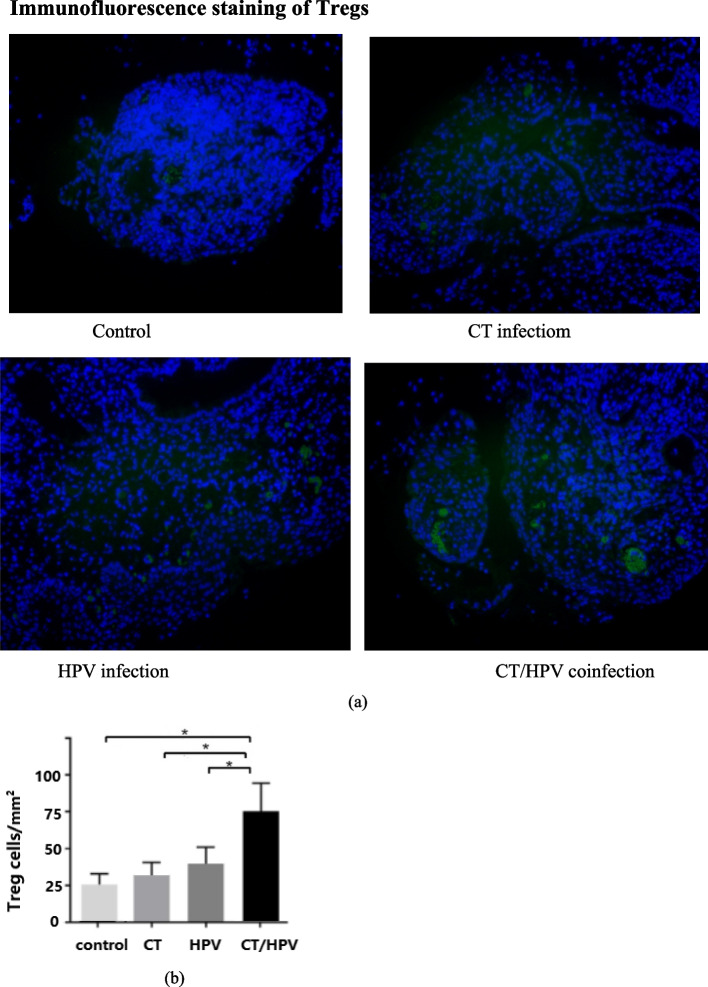
Immunofluorescence staining for FoxP3 (**a**) Representative image with the combined staining of DAPI in each group (*n* = 30 for each group). Scale bar, 75 µm. The arrow indicates FoxP3-positive cells. Green indicates Foxp3 + , blue indicates DAPI (**b**) infiltrating regulatory T cells (Tregs) in each group (*n* = 30 for each group). Differences between two groups were calculated with a one-way ANOVA, significant difference is indicated with asterisks. (*P < 0.05)

The number of FOXP3^+^ Tregs was increased in the HPV and CT groups compared with the control group. Comparison of the groups revealed a strong difference in Treg infiltration between the HPV and CT/HPV groups and between the CT and CT/HPV groups (*P* < 0.05). The CT/HPV group is most strongly infiltrated with Treg cells,, as shown in Fig. 5.

### Flow cytometry of T cell subsets and apoptosis

T-cell subsets and apoptosis were analysed in each group. Few or no CD4^+^ T cells and fewer CD8^+^ T cells were found in patients with CT/HPV coinfection, and there were significant differences in the frequency of CD4 + T cells between the HPV group and the CT/HPV group and between the HPV group and the control group. A significant difference was observed in the percentage of CD8 + cytotoxic T cells in the HPV group compared to that in the control group. In addition, there was no significant difference in the percentage of CD8 + cytotoxic T cells between the CT/HPV group and HPV group.

**Fig. 6:**
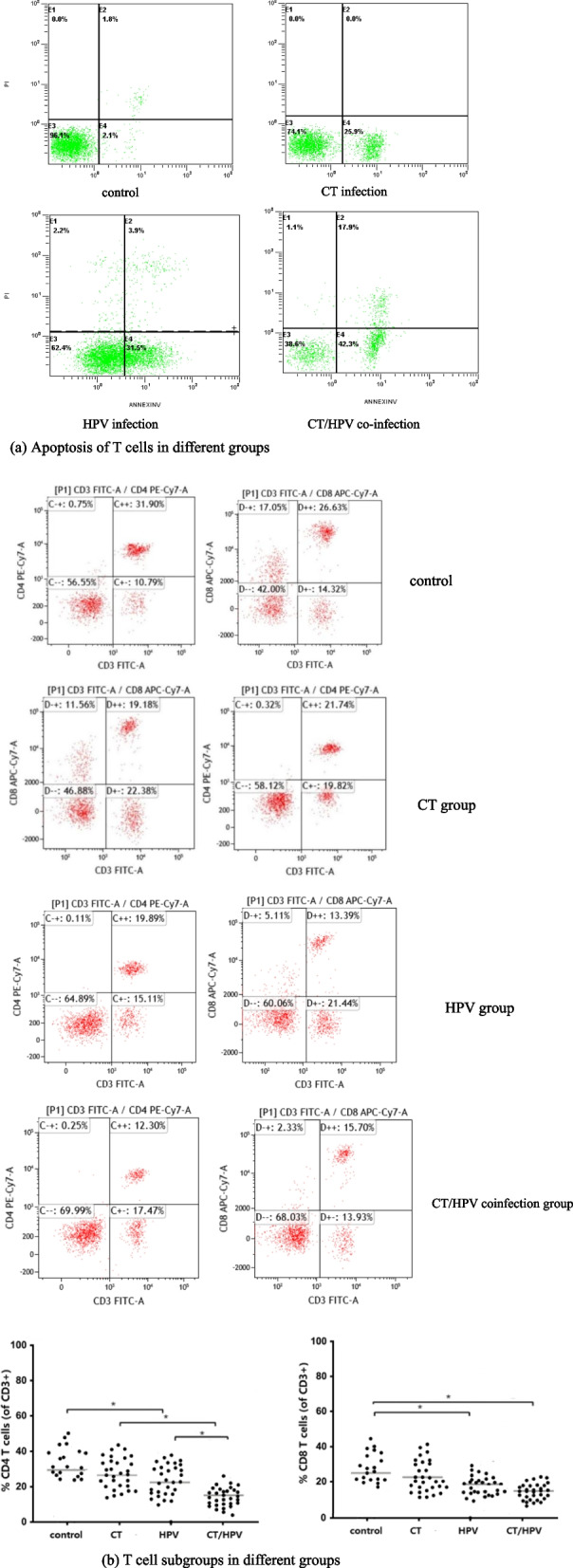
T-cell subsets and apoptosis in specimens from each group. **a** Apoptosis of T cells in different groups. **b** T-cell subgroups in different groups. The frequency of cervical CD4 + and CD8 + T cells is shown as the percentage of CD3 + lymphocytes. Each dot represents one patient. *N* = 30 for each population; differences were determined analysed using one-way ANOVA and Tukey’s test for median comparisons. * represents significant differences (*P* < 0.05)

The apoptosis of T cells in patients with CT/HPV coinfection was elevated compared to that in HPV infection alone and control subjects (Fig. 6).

## Discussion

Immune escape and persistence of hr-HPV infection are essential factors for the development of cervical cancer. Generally, most HPV infections are quickly eliminated by the host immune system, and cellular immunity plays a major role in this process [29].

HPV and *Chlamydia trachomatis* are the most common causes of sexually transmitted diseases worldwide. HPV and *Chlamydia trachomatis* share similar risk factors, such as younger age and higher number of sexual partners, CT/HPV coinfection rate increase rapidly in recent years [30].

In coinfection of CT with HPV, the persistent infection of HPV is enhanced [30, 31]. The co-infection with C. trachomatis and HPV (especially the 16, 18, 31, 33, 53, and 56 genotypes) is considered the most important risk factor for the presence of cervical cancer [32, 33].

In our research, CT/HPV coinfection patients usually have more serious precancerous lesions than HPV and CT groups,In patients with CT/HPV coinfection, the percentages of patients with LSIL and HSIL stage disease were 21.6% and 10.8%, respectively, which represented much higher rates than in the CT or HPV groups (*p* < 0.05), as shown in Table 1.

CT coinfection with HPV may modulate cervical cancer development. CT/HPV coinfection increased cell proliferation and altered mRNA expression patterns in cells that are involved in the mesenchymal phenotype [34, 35].This suggests that CT infection might promote HPV persistance and faciliates cervical cancer development.

Chronic CT infection can cause persistent HPV infection by reducing antigen-presenting cells and inhibiting cell-mediated immunity [31, 32].

A previous study showed that HPV-mediated suppression of LC immune function was a key mechanism by which HPV evades immune surveillance [6, 7]. Human LCs may become immunosuppressive after encountering HPV VLPs; however, this occurs in the absence of costimulation [36]. After HPV infection, LCs exhibit PI3K pathway and Akt activation and regulation of both the MAPK and NF-ĸB cascades [37], thereby modulating multiple signaling cascades involved in the regulation of immune responses. The PI3K pathway in LCs is activated by HPV infection, and CT infection can also activate the PI3K pathway, so CT/HPV coinfection results in further PI3K pathway activation.

After treatment with a PI3K inhibitor, LCs exhibited upregulated expression of surface costimulatory molecules and could initiate HPV-specific immune responses. As the inhibition of PI3K resulted in enhanced immunity [38], CT/HPV coinfection enhanced the activation of the PI3K pathway and inhibited the MAPK pathway, which further suppressed immune responses. This result indicates that CT causes persistent HPV infection by dysregulating the PI3K/Akt and MAPK pathways in LCs, and the activation of PI3K in LCs defines a mechanism of immune escape used by HPV.

In addition, HPV infection can reduce the LC number and antigen presentation ability compared to that of the control. TIn HPV infection, the lack of an effective immune response suggests that viral antigens are not adequately presented to T cells. CT/HPV coinfection significantly reduced the density and antigen-presenting abilities of LCs compared to HPV infection alone (*P* < 0.01). Only when HPV peptides presenting on the LC surface are transferred to T cells, will T cells not respond and may even become suppressed. The decreased LC quantity and antigen presentation ability caused by CT can result in worse local cellular immune function and persistent HPV infection, leading to atypical hyperplasia and cervical cancer.

CT/HPV coinfection also altered T-cell subsets. The results showed that patients with coinfection had few CD^4^ + T cells and fewer CD8^+^ cells and the CD4:CD8 ratios decreased to less than 1. Infiltrating Treg cells were present in the CT/HPV coinfection group. Additionally, CT/HPV coinfection exacerbated T-cell apoptosis.

CD8^+^ cytotoxic T lymphocyte (CTL)-mediated cytolysis of target cells plays an important role in the process of anti-HPV infection. The recognition of MHC I by CD8^+^ cells is a key step in the immune cytotoxicity of CD8^+^ CTLs. CD4^+^ T cells can also produce immunity against HPV infection, The immune effect may be activated after MHC II molecules are recognized by CD4^+^ T cells [39]. Compared with the HPV infection group, CT/HPV coinfection further downregulated the expression of MHC I and MHC II molecules on the LC surface and reduced the CD4^+^ and CD8^+^ CTL subsets, thus inhibiting cell-mediated immunity and leading to persistent HPV infection.

Regulatory T cells (Tregs) often appear in persistent HPV infection and are positively correlated with the lesion size [40]. The increased percentage of Tregs in this study was consistent with previous results.

In conclusion, CT infection exacerbates the activation of the PI3K/Akt pathway and the suppression of the MARK pathways during HPV infection, downregulates surface activation marker expression and decreases LC numbers,impairs the immune cytotoxicity of CD4^+^ and CD8^+^ T cells, and inhibits cell-mediated immunity and HPV clearance; these phenomena enhance persistent HPV infection, accelerate the natural process of HPV infection and lead to the development of precancerous lesions and cervical cancer.

Moreover, these findings suggest that in addition to a newly identified CT-enhanced immune escape mechanism used by HPV, PI3K may serve as a candidate target for inhibition to enhance HPV immunity.

### Supplementary Information

**Supplementary materials 1. **

**Supplementary materials 2. **

## Data Availability

The datasets that were used and/or analysed in the current study are available from the corresponding author upon reasonable request.
