# Supplementary material for: Chlamydia trachomatis enhances HPV persistence through immune modulation
Source: BMC Infect Dis. 2024 Feb 20;24:229. doi: 10.1186/s12879-024-09094-6 (PMC10880247; doi:10.1186/s12879-024-09094-6)
Supplement: Supplementary file 1 — Supplementary materials 1. [file 12879_2024_9094_MOESM1_ESM.pdf]

胶1

p-P13K (60kD)

CT/HPV CT HPV control control HPV CT CT/HPV control HPV CT CT/HPV

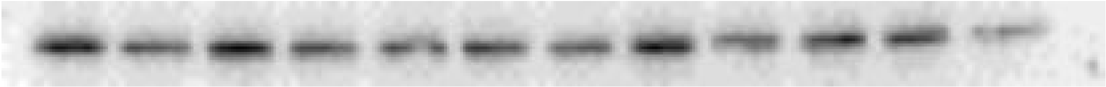

Cat:（ABCAM）ab278545
Blocking: 5%BSA, 1h
First Antibody: Anti-p- P13K (1:1000), 4℃，过夜
Second Antibody: HRP标记山羊抗兔
(1:5000)IgG(H+L), 1h
Expoure time:1s, 3s,10s,30s,60s.

胶2

p-AKT (60kD)

control HPV CT CT/HPV control HPV CT CT/HPV control HPV CT CT/HPV

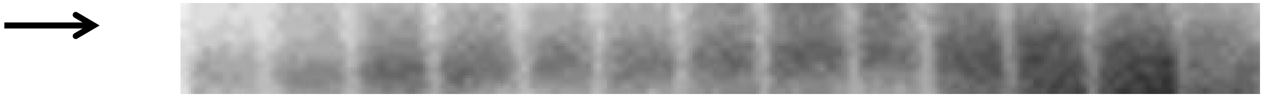

Cat:（CST）13038S
Blocking: 5%BSA, 1h
First Antibody: Anti- p-AKT (1:1000), 4°C，过夜
Second Antibody: HRP标记山羊抗兔
(1:5000)IgG(H+L), 1h
Expoure time:1s, 3s,10s,30s,60s.

胶3

AKT (59kD)

control HPV CT CT/HPV control HPV CT CT/HPV control HPV CT CT/HPV

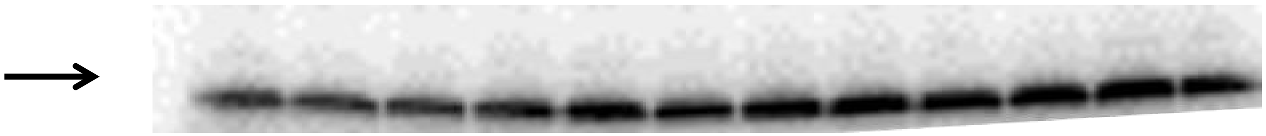

Cat:（CST）9272S
Blocking: 5%BSA, 1h
First Antibody: Anti- AKT(1:1000), 4°C，过夜
Second Antibody: HRP标记山羊抗兔
(1:5000)IgG(H+L), 1h
Expoure time:1s, 3s,10s,30s,60s.

胶4

GAPDH (37kD)

control HPV CT CT/HPV control HPV CT CT/HPV control HPV CT CT/HPV

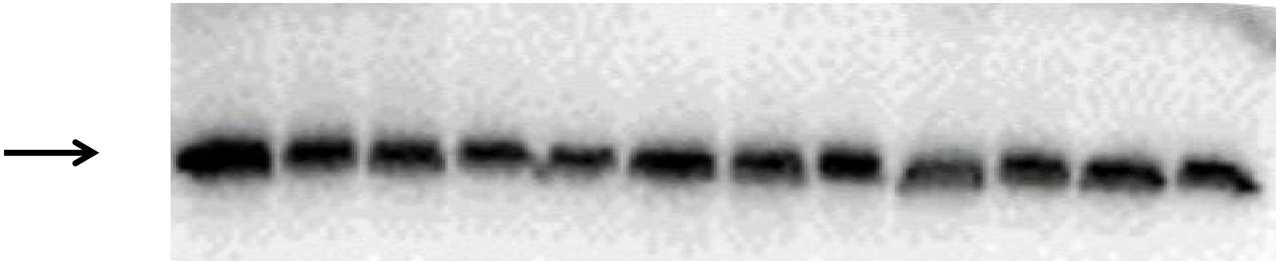

Cat:（CST）5174T
Blocking: 5%BSA, 1h
First Antibody: Anti- GAPDH (1:1000), 4℃，过夜
Second Antibody: HRP标记山羊抗兔
(1:5000)IgG(H+L), 1h
Expoure time:1s, 3s,10s,30s,60s.

胶5

ERK1/2(44KD)

control HPV CT CT/HPV control HPV CT CT/HPV control HPV CT CT/HPV

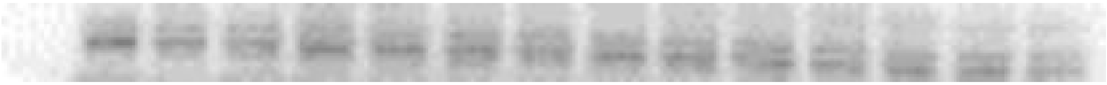

Cat:（CST）4695S
Blocking: 5%BSA, 1h
First Antibody:ERK1/2 (1:1000), 4°C，过夜
Second Antibody: HRP标记山羊抗兔
(1:5000)IgG(H+L), 1h
Expoure time:1s, 3s,10s,30s,60s.

胶6

MKK4(44kD)

control HPV CT CT/HPV control HPV CT CT/HPV control HPV CT CT/HPV

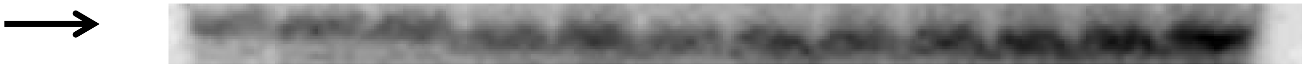

Cat:（CST）9152S
Blocking: 5%BSA, 1h
First Antibody: Anti- MKK4(1:1000), 4°C，过夜
Second Antibody: HRP标记山羊抗兔
(1:5000)IgG(H+L), 1h
Expoure time:1s, 3s,10s,30s,60s.

胶7

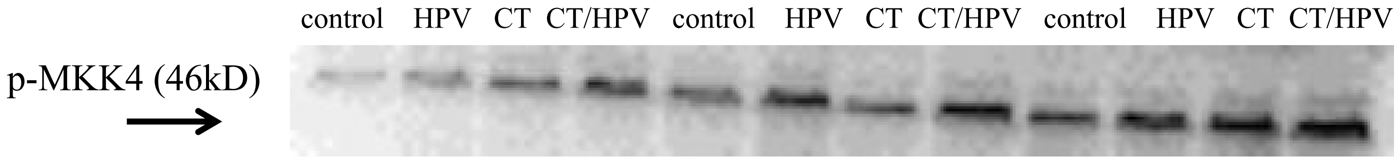

Cat:（CST）9151S
Blocking: 5%BSA, 1h
First Antibody:p-MKK4(1:1000), 4℃，过夜
Second Antibody: HRP标记山羊抗兔
(1:5000)IgG(H+L), 1h
Expoure time:1s, 3s,10s,30s,60s.

胶8

control HPV CT CT/HPV control HPV CT CT/HPV control HPV CT CT/HPV

GAPDH (37kD) →

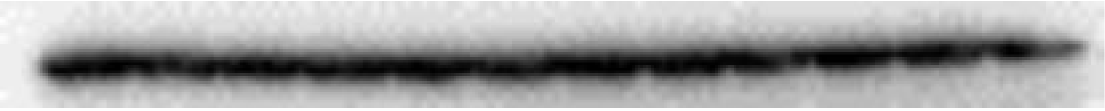

Cat:（CST）5174T
Blocking: 5%BSA, 1h
First Antibody:GAPDH (1:1000), 4℃，过夜
Second Antibody: HRP标记山羊抗兔
(1:5000)IgG(H+L), 1h
Expoure time:1s, 3s,10s,30s,60s.

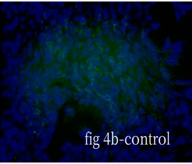
fig 4b-control

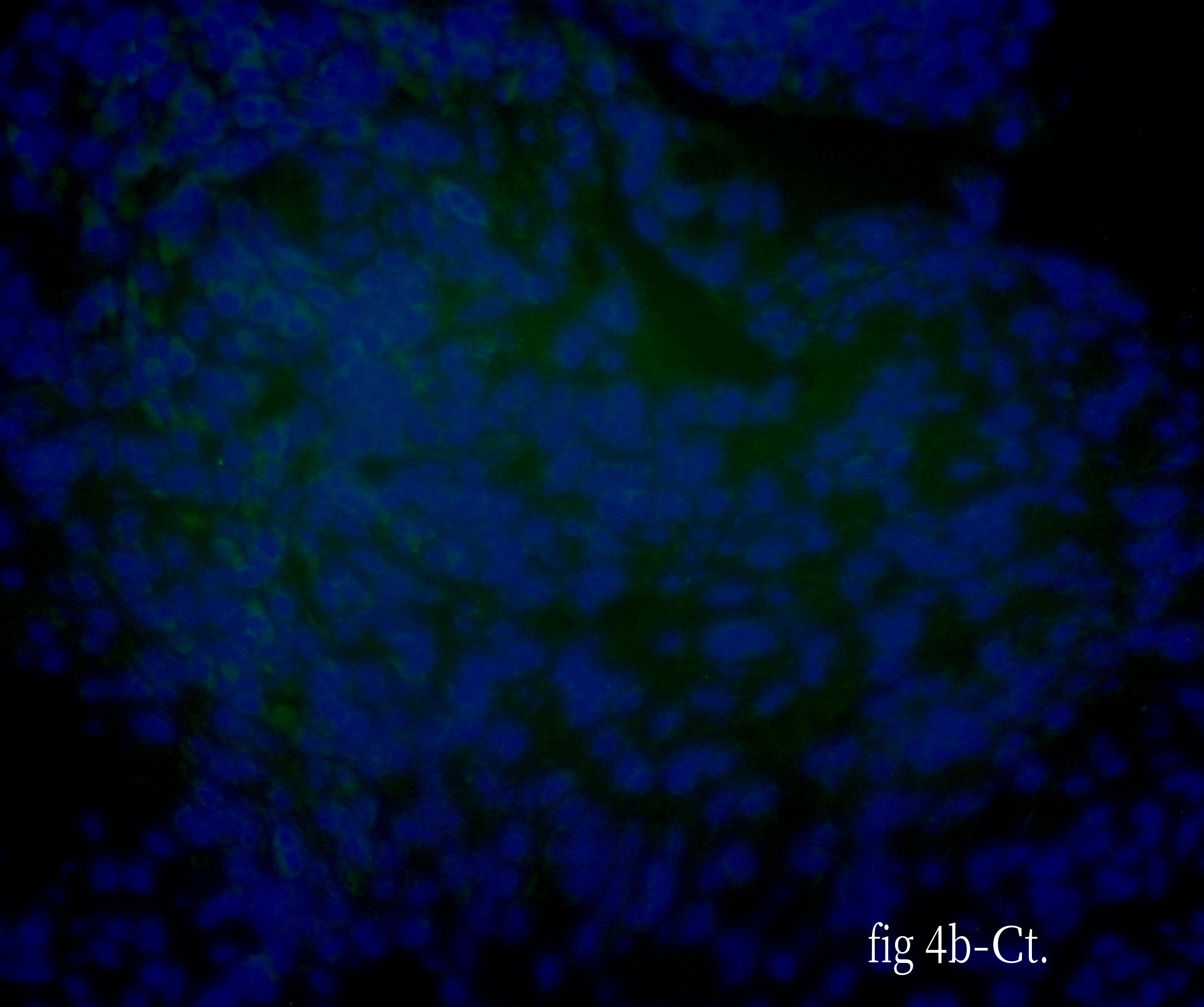

fig 4b-Ct.

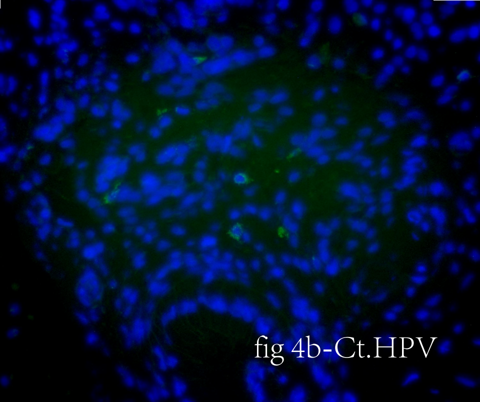
fig 4b-Ct.HPV

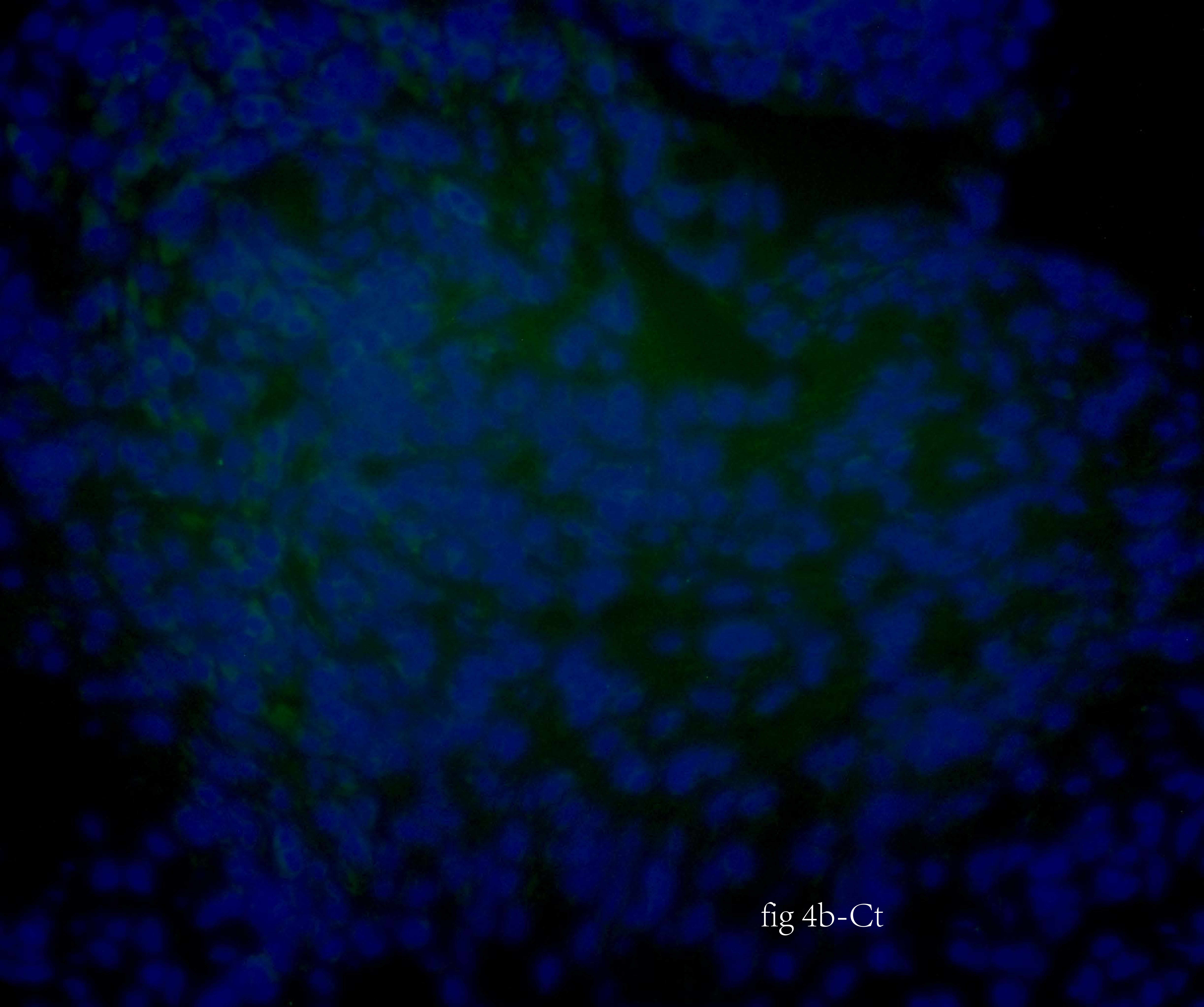
fig 4b-Ct

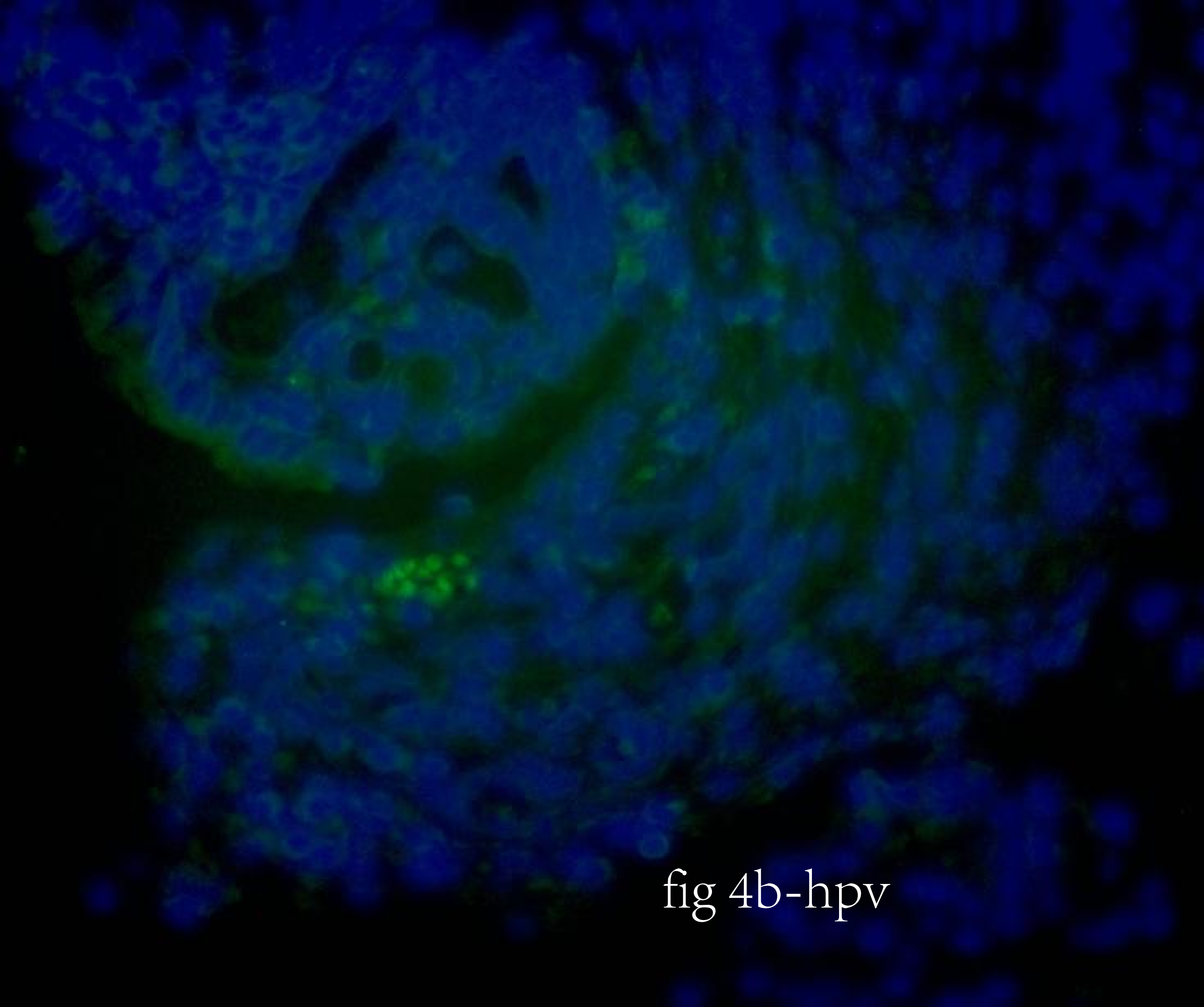
fig 4b-hpv

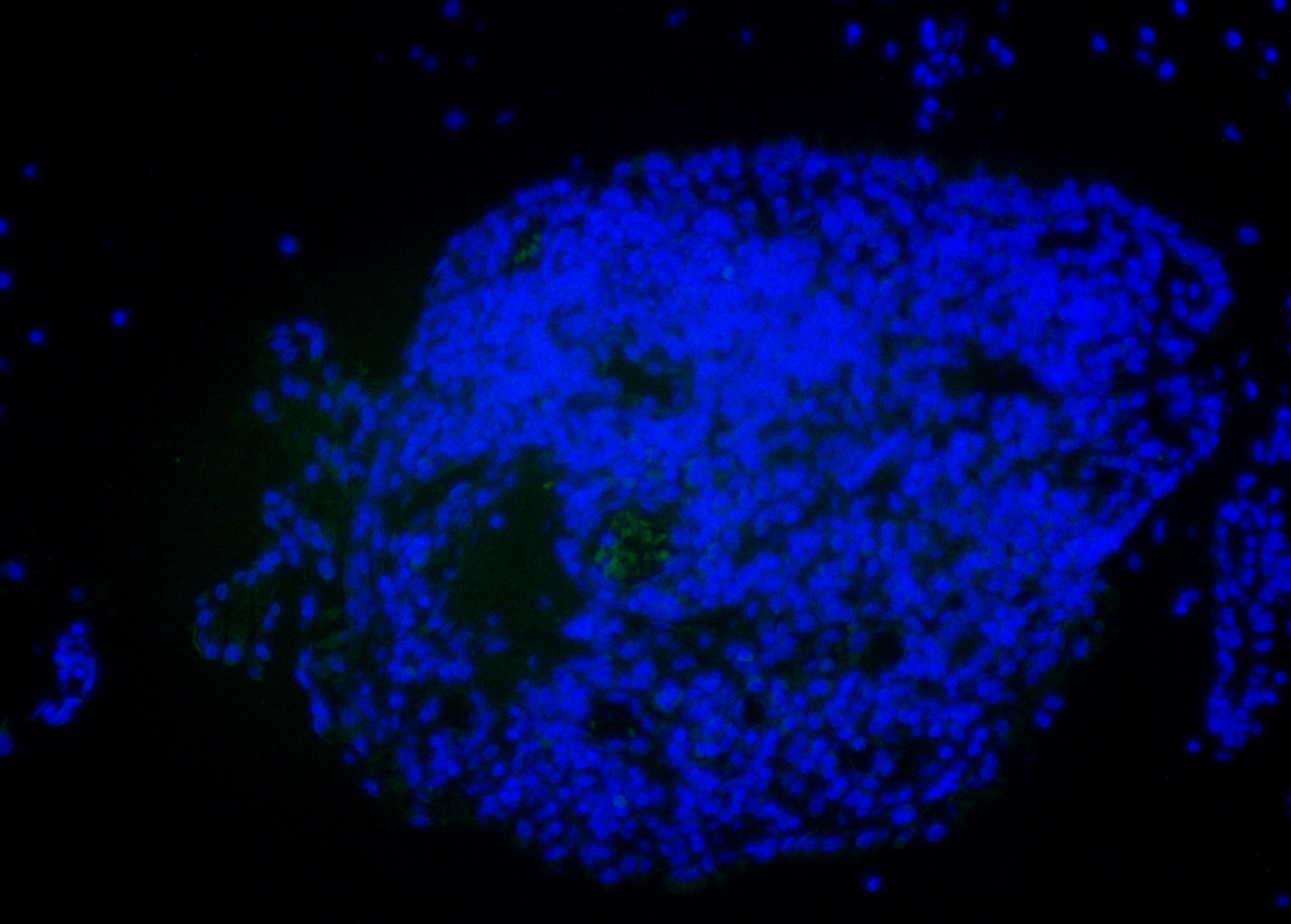

fig 5a-control

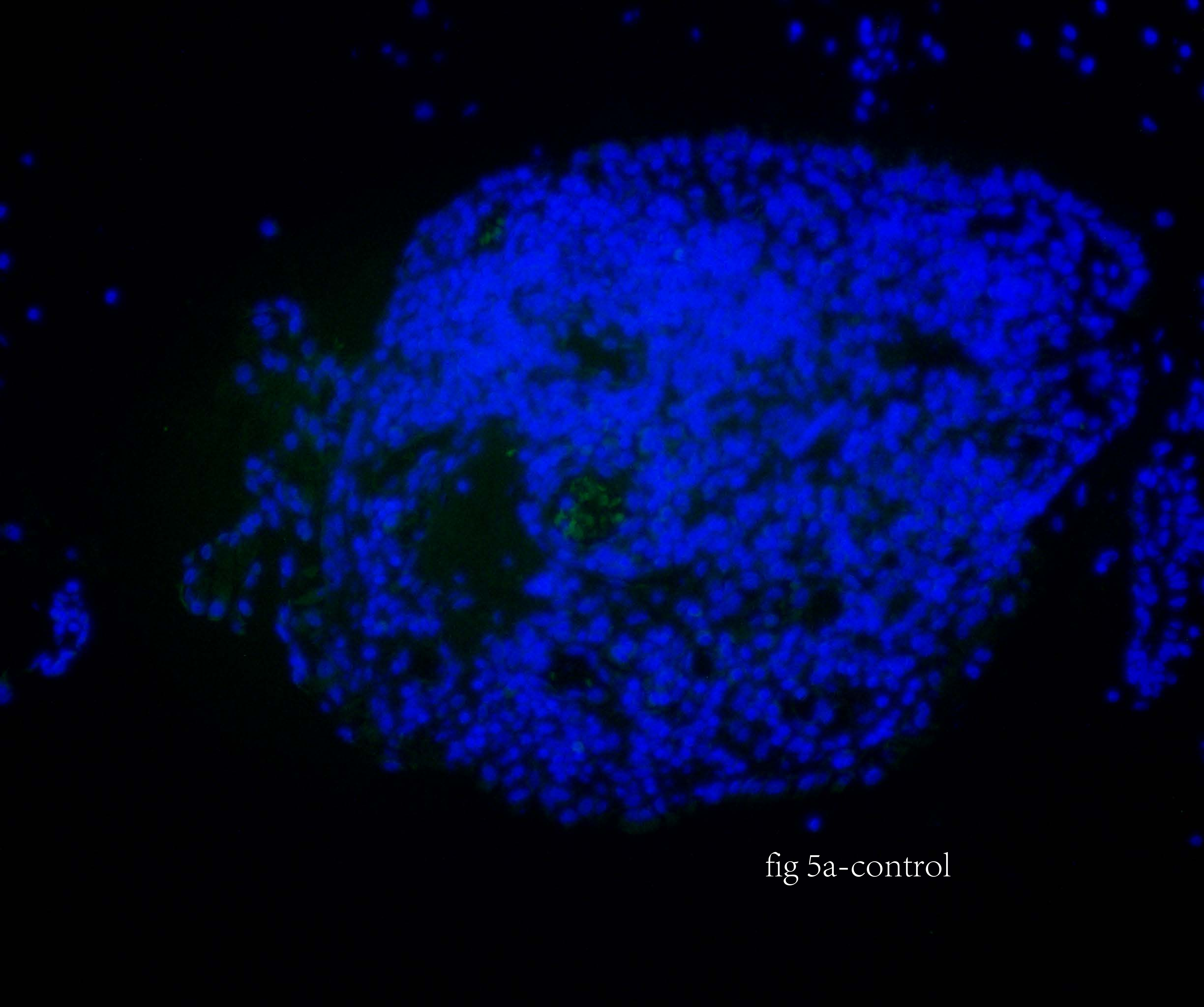

fig 5a-control

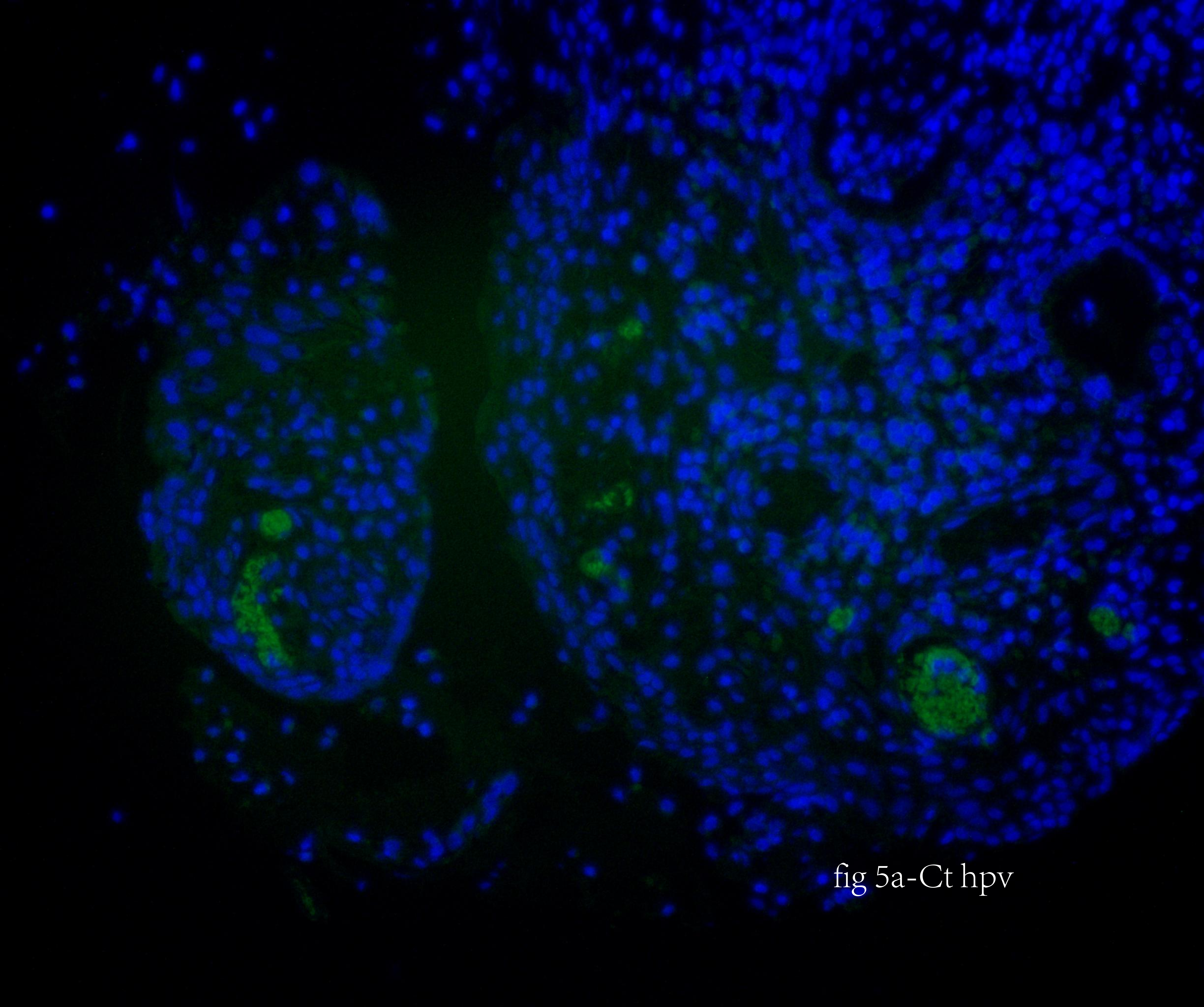

fig 5a-Ct hpv

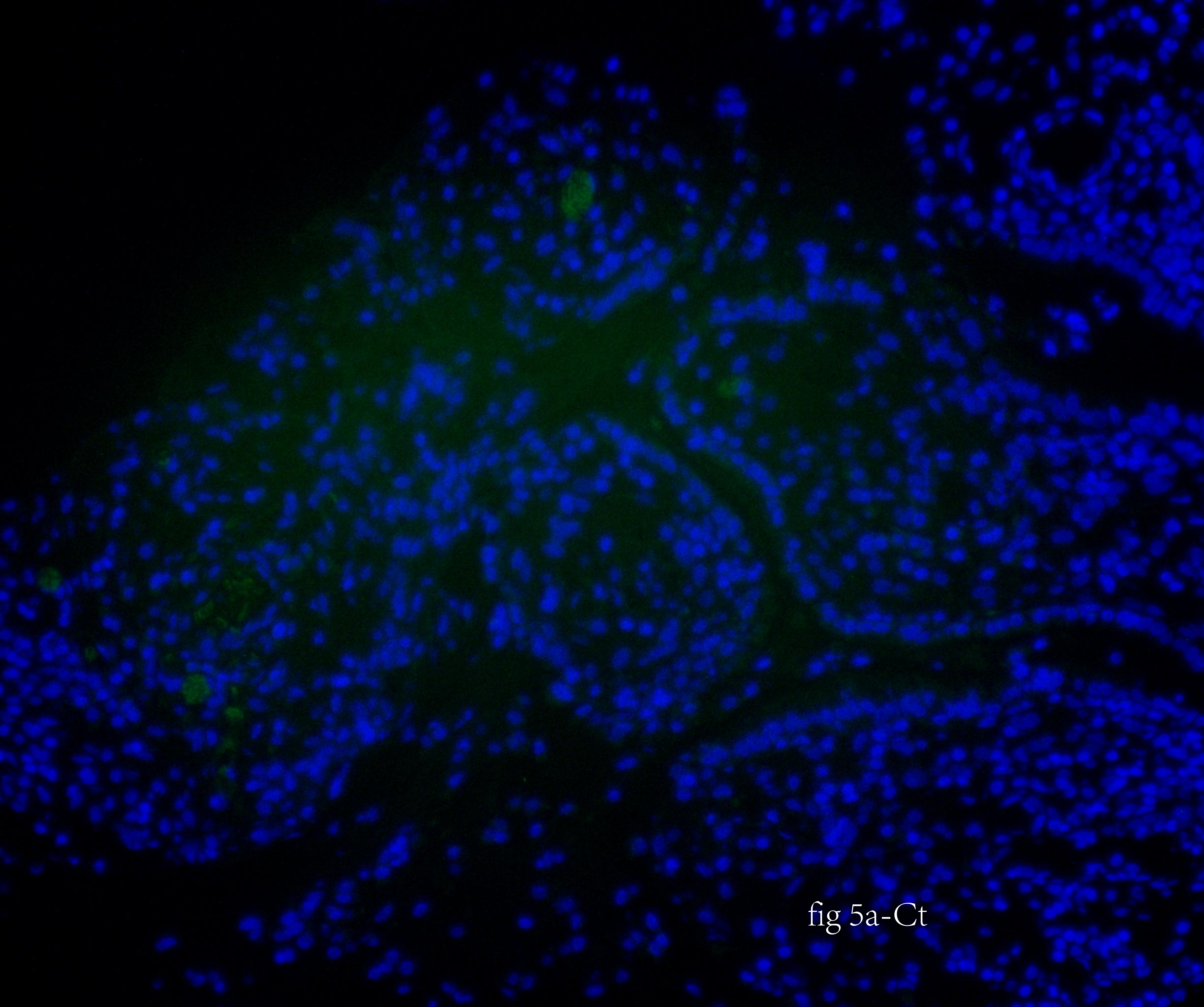
fig 5a-Ct

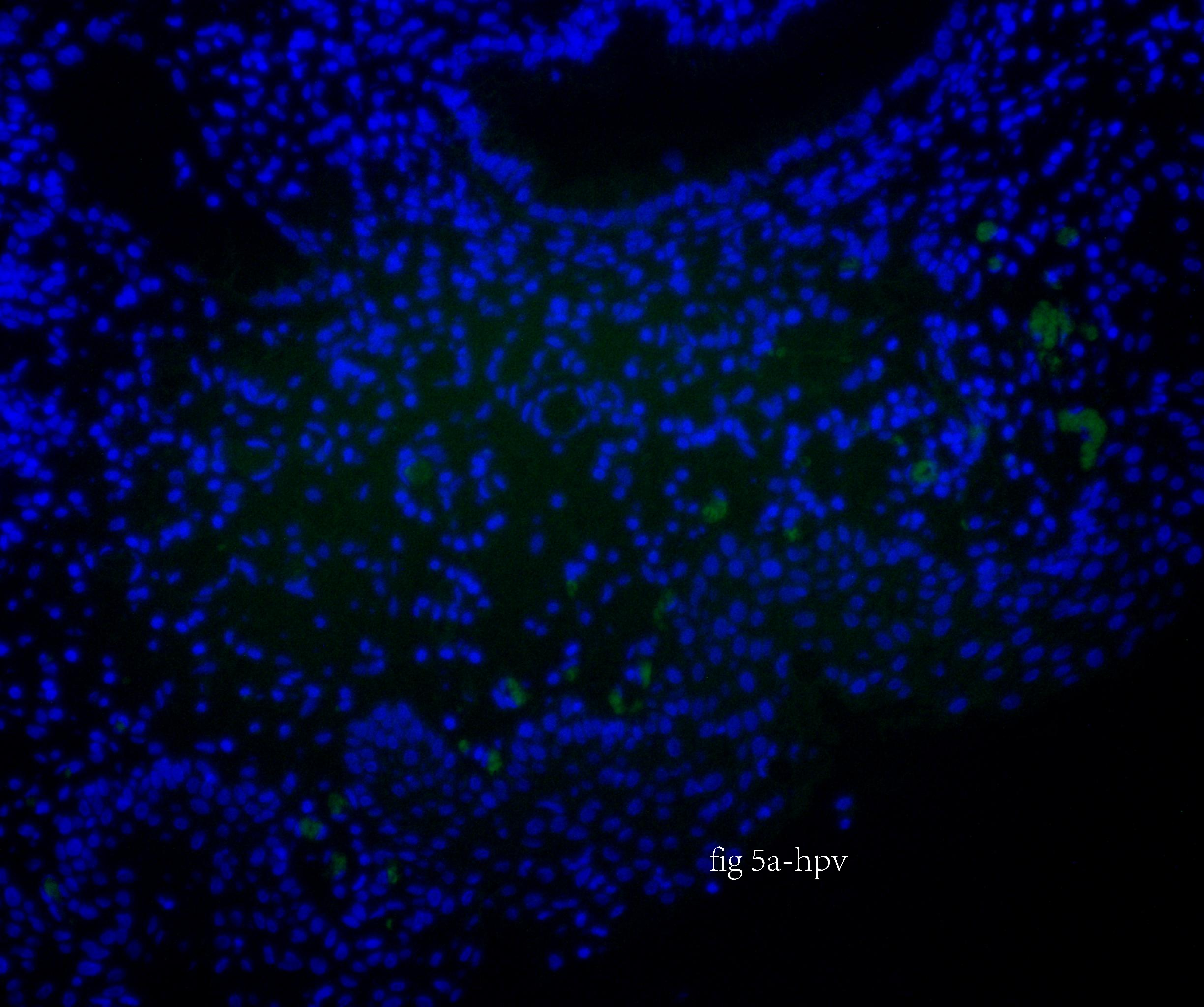
fig 5a-hpv

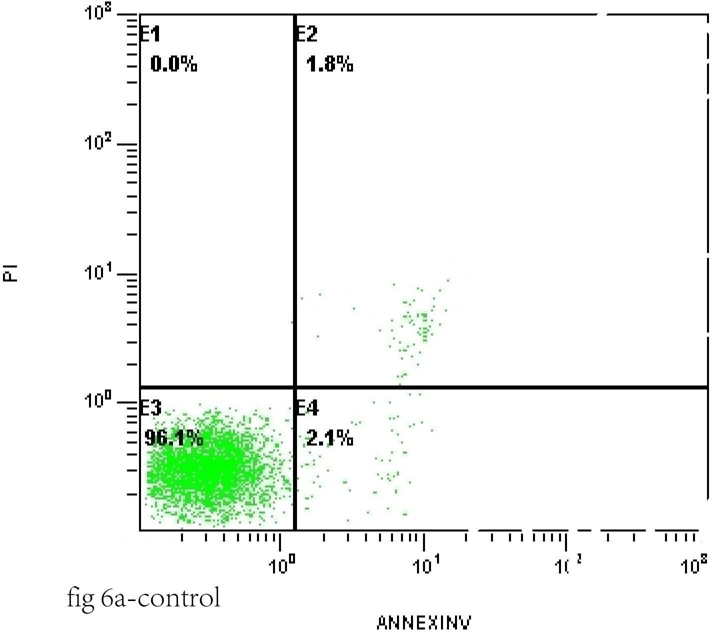

fig 6a-control

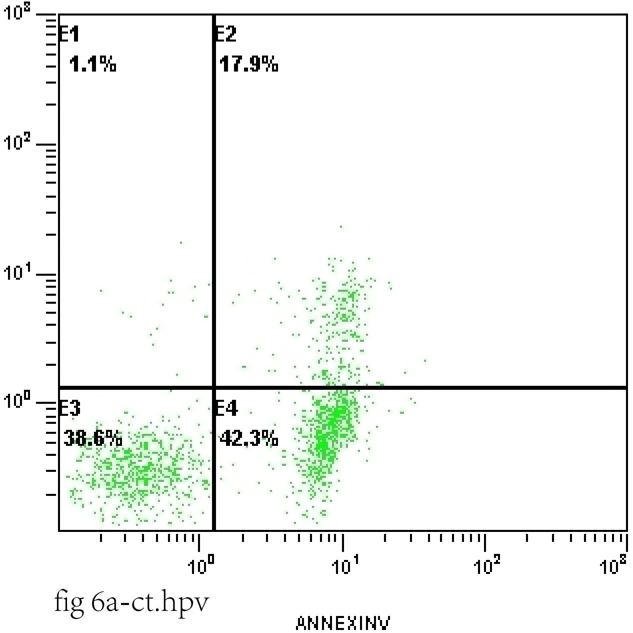

fig 6a-ct.hpv

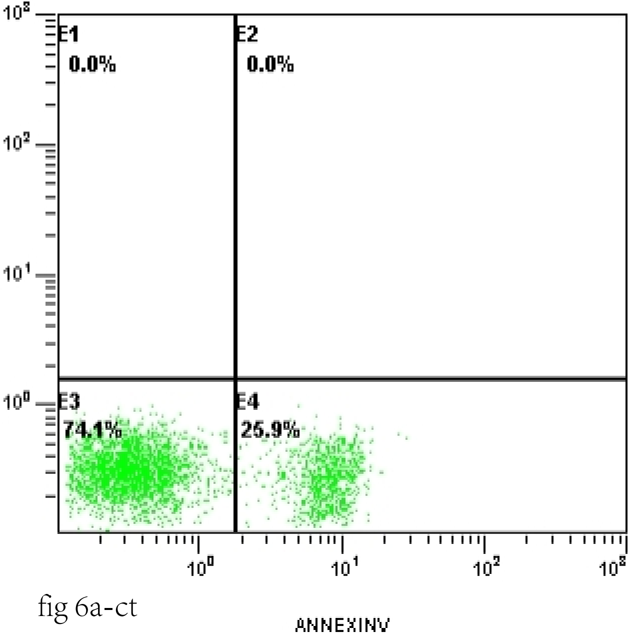

fig 6a-ct

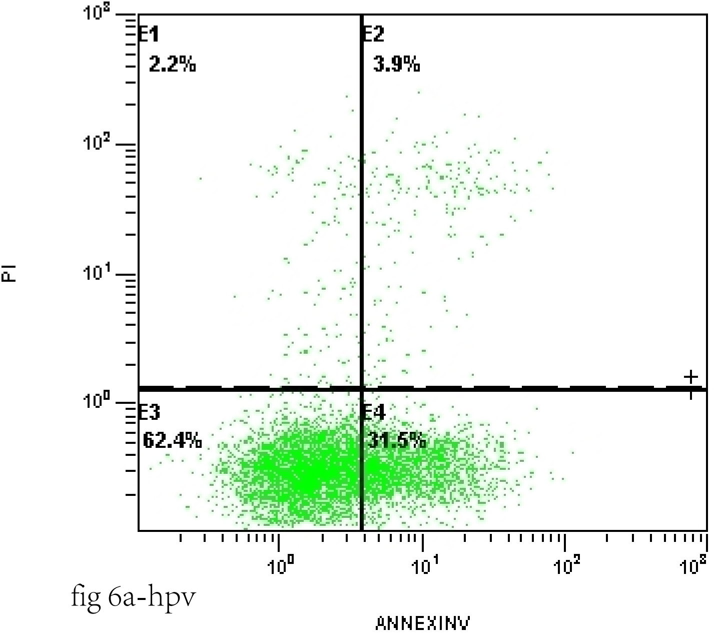

fig 6a-hpv

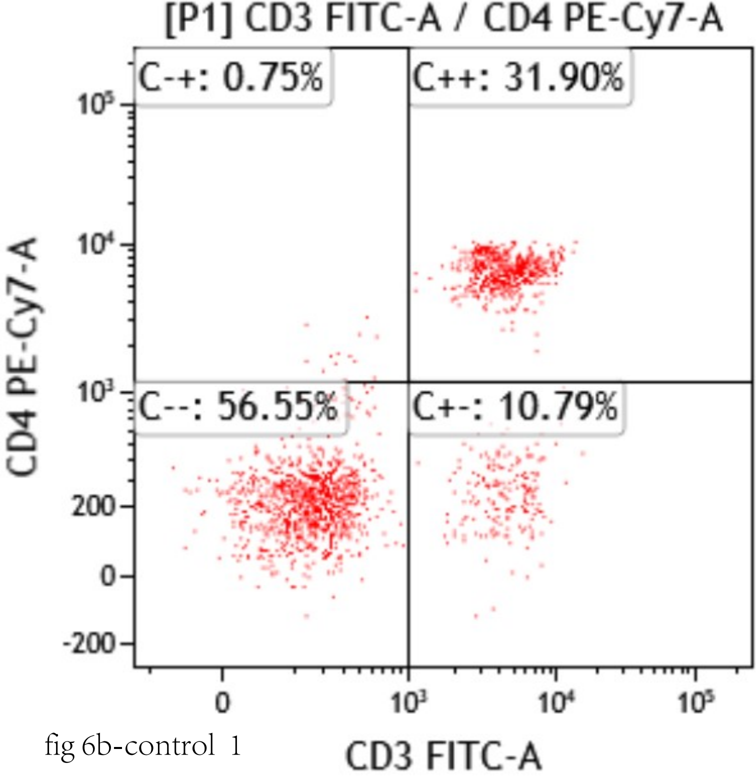

fig 6b-control 1

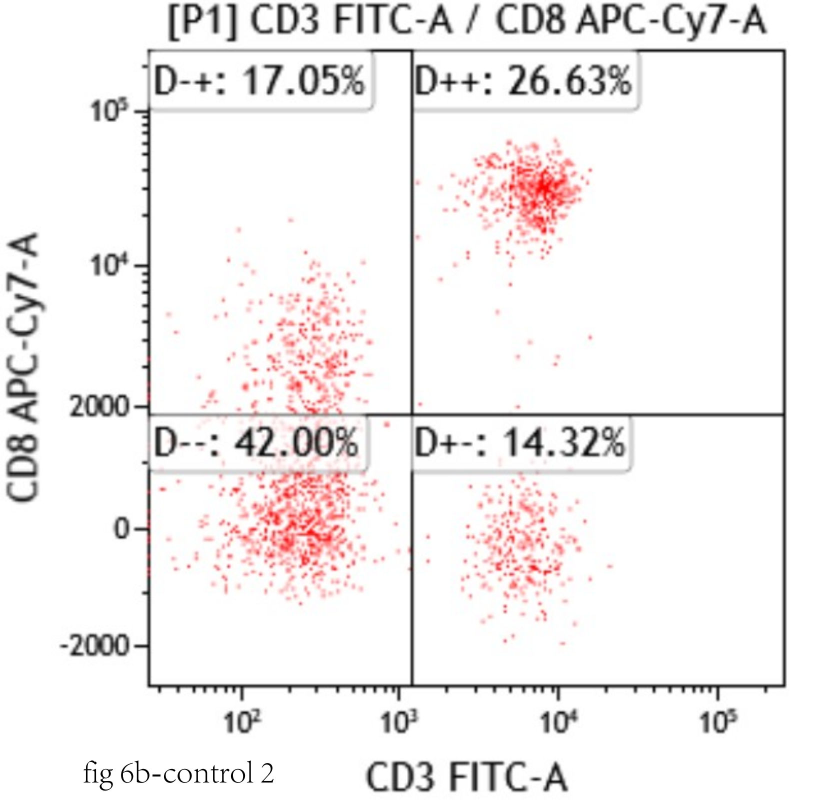

fig 6b-control 2

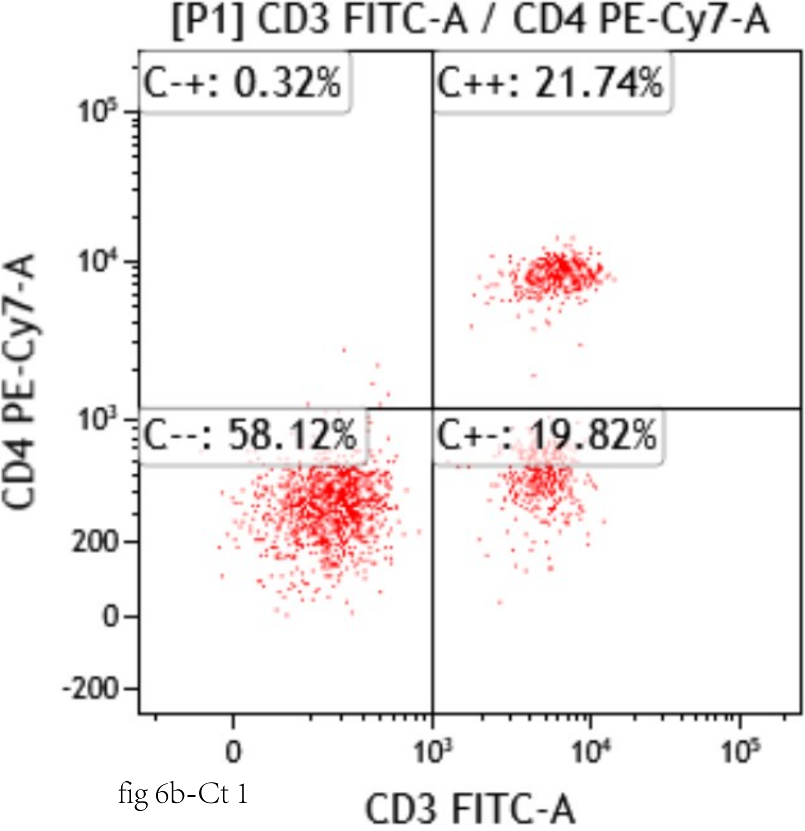
[P1] CD3 FITC-A / CD4 PE-Cy7-A
C-+: 0.32%
C++: 21.74%
C--: 58.12%
C+-: 19.82%
CD4 PE-Cy7-A
CD3 FITC-A
10^5
10^4
10^3
200
0
-200

fig 6b-Ct 1

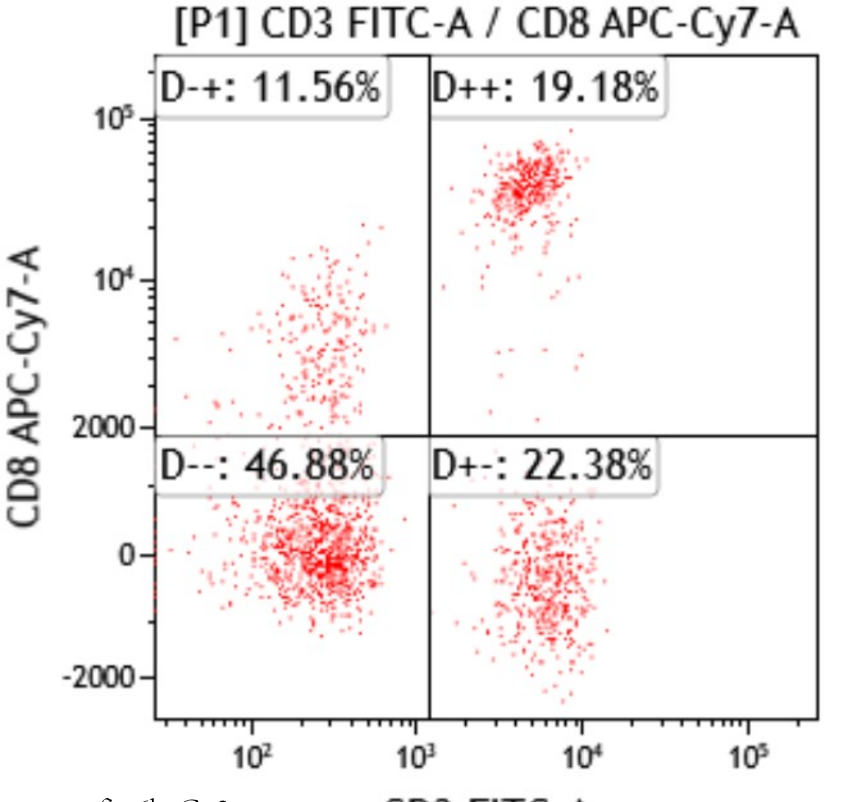
[P1] CD3 FITC-A / CD8 APC-Cy7-A
D-+: 11.56%
D++: 19.18%
D--: 46.88%
D+-: 22.38%
CD8 APC-Cy7-A
CD3 FITC-A

fig 6b-Ct 2

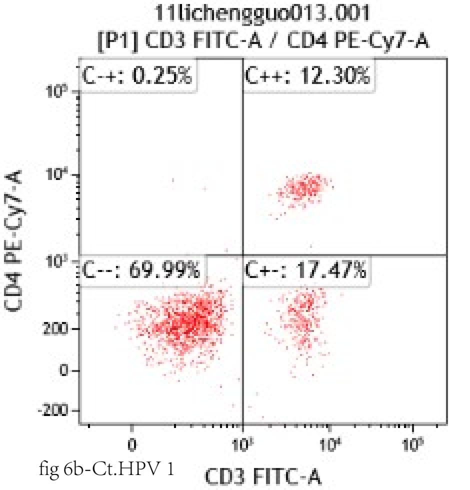

fig 6b-Ct.HPV 1

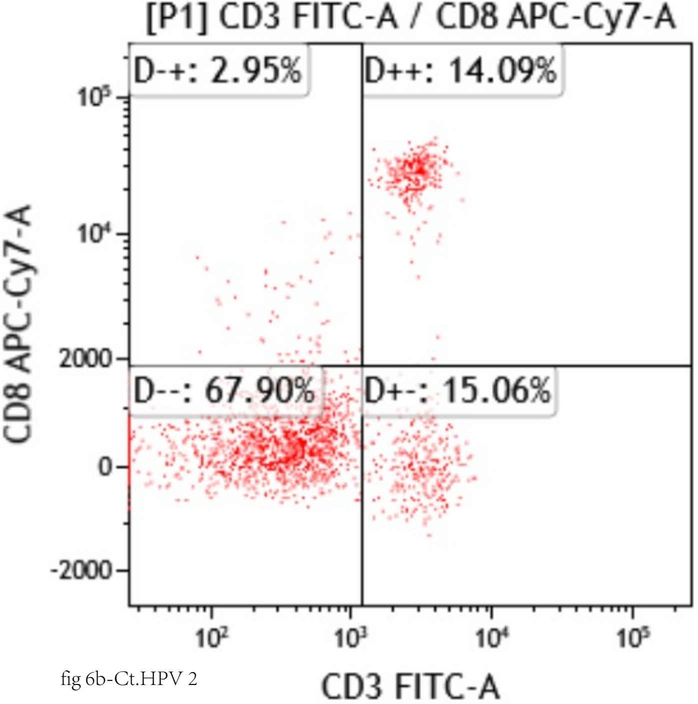

fig 6b-Ct.HPV 2

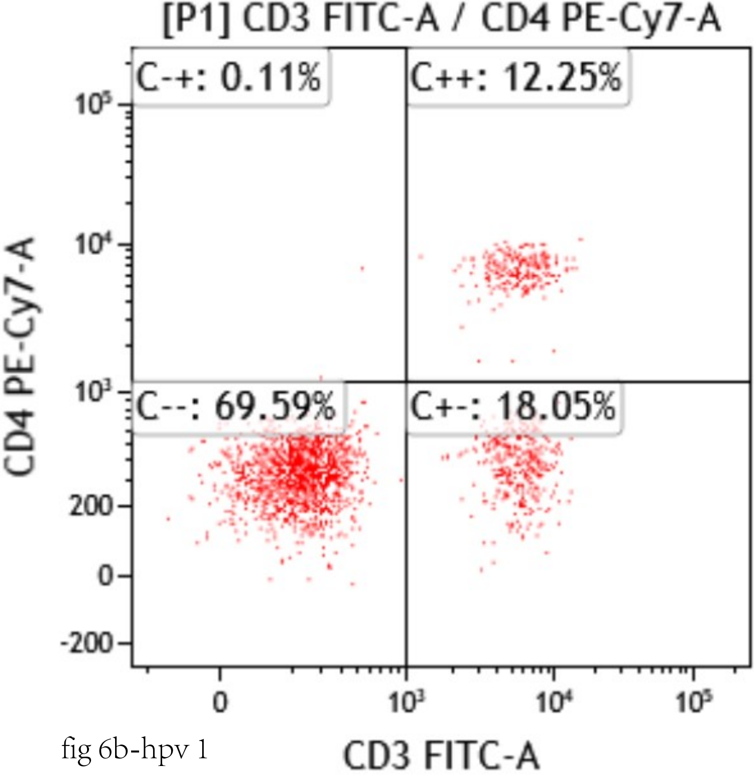
[P1] CD3 FITC-A / CD4 PE-Cy7-A
C-+: 0.11%
C++: 12.25%
C--: 69.59%
C+-: 18.05%
CD4 PE-Cy7-A
CD3 FITC-A
-200
0
200
10^3
10^4
10^5

fig 6b-hpv 1

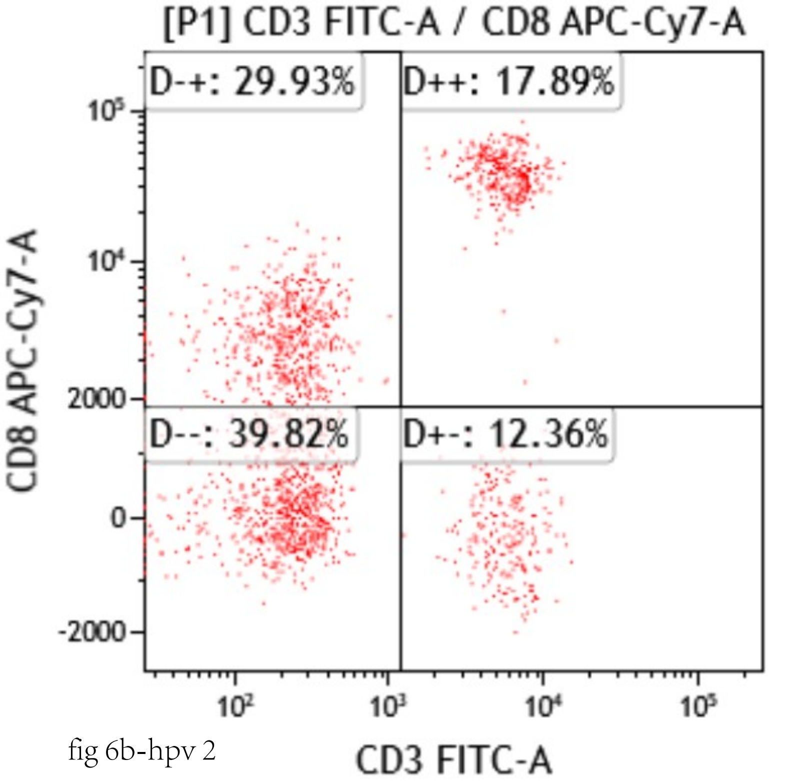
[P1] CD3 FITC-A / CD8 APC-Cy7-A
D-+: 29.93%
D++: 17.89%
D--: 39.82%
D+-: 12.36%
CD8 APC-Cy7-A
CD3 FITC-A

fig 6b-hpv 2
