# Supplementary figures and images for: Chlamydia trachomatis enhances HPV persistence through immune modulation
Source: BMC Infect Dis. 2024 Feb 20;24:229. doi: 10.1186/s12879-024-09094-6 (PMC10880247; doi:10.1186/s12879-024-09094-6)

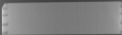

2022-08-26

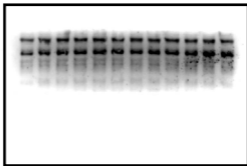

erk1/2

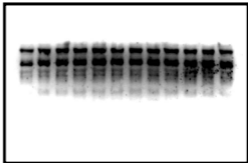

erk1/2

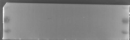

2022-08-25

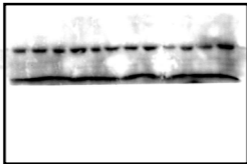

p-AKT

100 200 300 400 500 600 700 800 900 1000

100 200 300 400 500 600 700 800 900 1000

PI3K 10S

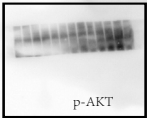

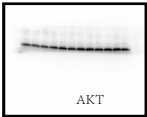

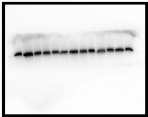

GAPDH

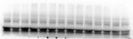

ERK1/2

1998

MKK4 10S

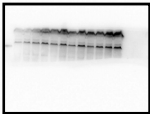

p-MKK4 10S

Supplement: Supplementary file 2 — Supplementary materials 2. [file 12879_2024_9094_MOESM2_ESM.pdf]
